# Direct Electrodeposition of Bimetallic Nanostructures on Co-Based MOFs for Electrochemical Sensing of Hydrogen Peroxide

**DOI:** 10.3389/fchem.2022.856003

**Published:** 2022-03-11

**Authors:** Yixuan Xie, Xianhua Shi, Linxi Chen, Jing Lu, Xiange Lu, Duanping Sun, Luyong Zhang

**Affiliations:** ^1^ Center for Drug Research and Development, Guangdong Provincial Key Laboratory of Pharmaceutical Bioactive Substances, Guangdong Pharmaceutical University, Guangzhou, China; ^2^ National and Local United Engineering Lab of Druggability and New Drugs Evaluation, School of Pharmaceutical Sciences, Sun Yat-Sen University, Guangzhou, China; ^3^ New Drug Screening Center, Jiangsu Center for Pharmacodynamics Research and Evaluation, China Pharmaceutical University, Nanjing, China

**Keywords:** electroanalysis, bimetallic nanoparticles, electrochemical sensor, hydrogen peroxide, cancer cells

## Abstract

Hydrogen peroxide (H_2_O_2_) is the most significant reactive oxygen species in biological systems. Here, we reported an electrochemical sensor for the detection of H_2_O_2_ on the basis of bimetallic gold-platinum nanoparticles (Au_3_Pt_7_ NPs) supported by Co-based metal organic frameworks (Co-MOFs). First, Au_3_Pt_7_ NPs, with optimal electrocatalytic activity and accessible active surface, can be deposited on the surface of the Co-MOF–modified glassy carbon electrodes (Au_3_Pt_7_/Co-MOFs/GCE) by one-step electrodeposition method. Then, the electrochemical results demonstrated that the two-dimensional (2D) Co-MOF nanosheets as the supporting material displayed better electrocatalytic properties than the 3D Co-MOF crystals for reduction of H_2_O_2_. The fabricated Au_3_Pt_7_/2D Co-MOF exhibited high electrocatalytic activity, and the catalytic current was linear with H_2_O_2_ concentration from 0.1 μM to 5 mM, and 5–60 mM with a low detection limit of 0.02 μM (S/N = 3). The remarkable electroanalytical performance of Au_3_Pt_7_/2D Co-MOF can be attributed to the synergistic effect of the high dispersion of the Au_3_Pt_7_ NPs with the marvelous electrochemical properties and the 2D Co-MOF with high-specific surface areas. Furthermore, this sensor has been utilized to detect H_2_O_2_ concentrations released from the human Hela cells. This work provides a new method for improving the performance of electrochemical sensors by choosing the proper support materials from diverse crystal morphology materials.

## Introduction

Hydrogen peroxide (H_2_O_2_), as one of the most significant reactive oxygen species (ROS), commonly exists in biological processes as a ubiquitous intracellular messenger or receptor signaling in various cells (Ushio-Fukai et al., 1999). On the one hand, H_2_O_2_ was considered as a toxic by-product during the process of aerobic metabolism (Rhee et al., 2005). On the other hand, some serious human diseases will be triggered by an abnormal concentration level of H_2_O_2_ (Trachootham et al., 2009; Yang et al., 2011), including myocardial infarct (Griendling, 2004), Alzheimer’s disease (Markesbery, 1997), aging (Giorgio et al., 2007; Hayyan et al., 2016), and cancers (Jorgenson et al., 2013). Therefore, developing an accurate and sensitive detection method to monitor the H_2_O_2_ concentration at the cellular level is vital and urgent for future clinic diagnosis and cancer treatment (Dong et al., 2019a). Until now, various analytical technologies have been utilized to detect H_2_O_2_, such as fluorescence (Ren et al., 2016; Ma et al., 2017), chemiluminescence (Yuan and Shiller, 1999; Xie and Huang, 2011), spectrophotometry (Yu et al., 2017), chromatography (Gimeno et al., 2015), and electrochemistry. Compared with the established analytical methods, the electrochemical sensing technique is a promising approach to attain dynamic analysis of H_2_O_2_ concentration for its outstanding properties, specifically high sensitivity, good selectivity, simple operation, and low cost.

Electrochemical sensors for testing H_2_O_2_ can be subdivided into natural enzymes and nanomaterial-based artificial enzymes (nanozymes)–based sensors. The natural enzymes were known as peroxidases, such as horseradish peroxidase (Sun et al., 2004). The enzyme-based electrochemical sensors have been widely utilized in electroanalysis for the advantage of specificity and fast response to the target substance. However, natural enzymes are restricted to their inherent weaknesses, including the uncontrollable deactivation and hardship in purification (Wang et al., 2018). Therefore, to overcome the shortages of natural enzymes, nanozymes have been taken into consideration as robust alternatives. Until now, a series of nanomaterials have been proved to possess intrinsic peroxidase-mimicking catalytic properties (Wu et al., 2019), including noble metals, metal oxides, and carbon-based nanomaterials (Wei and Wang, 2013).

Among various enzyme mimics, bimetallic nanoparticles (NPs) have been widely employed to fabricate electrochemical biosensors. It usually performed better electrocatalytic properties than its monometallic counterparts (Iyyamperumal et al., 2013; Li et al., 2020; Yang et al., 2021). As previously reported, the properties of electrons can be improved by the synergistic effects of each monometallic nanomaterials (Renner et al., 2006). However, few limitations are constraining the further application of these bimetallic particles-based electrochemical sensors: 1) it is usually a challenge to meet the satisfactory sensitivity of the living cells detection; and 2) their thermodynamic instability and tendency to aggregate, owing to their high surface free energy (Wang et al., 2019).

These disadvantages can be improved by immobilizing metal NPs in/on supporting structures. Recently, metal organic frameworks (MOFs) formed by the self-assembly of organic ligands and metal ions (Batten et al., 2013) have drawn extensive attention as supporting materials because of their inherent advantages (Furukawa et al., 2013). Among various MOFs, Co-based MOFs were widely used in fabricating electrochemical sensors as supporting material (Dong et al., 2019b). According to the previous research, the zeolite imidazolate framework (ZIF)–67 which compounded of the imidazole-based organic linkers and transition metal (Co) displaying a desirable 3D structure rhombic dodecahedron morphology, and it has been widely utilized for fabricating the 3D Co-MOF–modified electrodes. Lu et al. (2020) constructed a novel electrochemical sensor with the help of 3D Co-MOF to load artificial enzymes Ag nanostructures. Recently, two-dimensional (2D) Co-MOF has drawn extensive attention as supporting material in fabricating electrochemical sensors. The meso-tetra-(4-carboxyphenyl)–substituted porphyrins (TCPP) is the excellent ligand for the constructing 2D MOF due to their tetragonal symmetry and rigid structure (Zhao et al., 2018a). When the TCPP ligands connect with Co^2+^, 2D Co-MOF nanosheets was formed. Four Co paddle-wheel metal nodes link one TCPP ligand. These TCPP linked ultrasmall 2D metalloporphyrinic MOF nanosheets have been used to fabricating electrochemical sensors for electrochemical catalyzing and sensing applications (Zhao et al., 2015). For example, Huang et al. (2017) reported a novel sensor for catalyzing the cascade reactions. The electrochemical sensor was fabricated by peroxidase mimics material Au NPs and artificial glucose oxidase, ultrasmall 2D metalloporphyrinic MOF nanomaterial. Ma et al. (2019) reported a novel electrochemical sensor fabricated by TCPP-based 2D Cu-MOF nanosheets and Ag NPs. Because of the smaller size and more accessible active sites (Xu et al., 2016; Zhao et al., 2018b), 2D Co-MOF may combine other functionalized materials better than 3D bulk MOF crystals (Tan et al., 2017; Liu et al., 2020). However, which morphology of MOF can perform better in loading artificial enzymes that still need more exploration?

**SCHEME 1:**
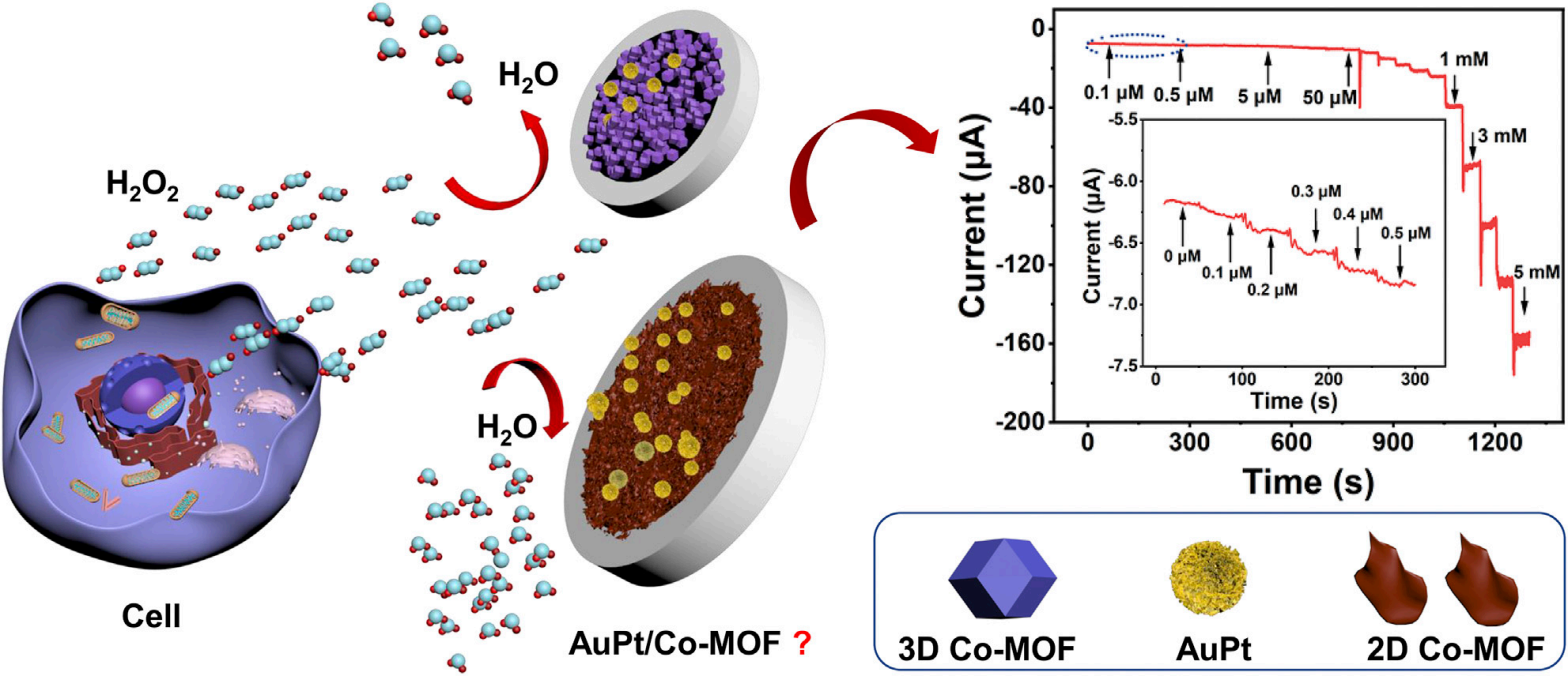
The electrochemical sensor for detecting H_2_O_2_ secreted from HeLa cells in response to drug stimulation.

In this work, our experiments first tried to answer whether AuPt NPs are a much better catalyst for H_2_O_2_ reduction reaction than either Au or Pt NPs. Then, we evaluated the electrocatalytic activity of 3D and 2D Co-MOF–modified electrodes, figuring out the influences of morphology for a supporting material. During the electrodeposition process with a negative voltage, AuPt NPs can be easily reduced from the solution adhering to the surface of the working electrode steadily (Wirtz and Martin, 2003). After comparing a series ration of Au NPs and Pt NPs with (V:V = 1:9, 3:7, 5:5, 7:3, and 9:1), a novel sensor was constructed which bimetallic Au_3_Pt_7_ NPs decorated on the 2D Co-MOF–modified glassy carbon electrode (Au_3_Pt_7_/2D Co-MOFs/GCE) by electrodeposition. This high-performance electrochemical sensor was successfully utilized to monitor H_2_O_2_ concentration in real-time, and it performed a desirable property for tracing H_2_O_2_ in human cancer samples. Scheme 1 illustrated the detecting process of in suit analyzing of H_2_O_2_ secreted from Hela cells with drug stimulation by electrochemical sensor.

## Experimental

### Reagents and Apparatus

The reagents and apparatus were provided in detail in the Supporting Information.

### Synthesis of 2D and 3D Co-MOF

In this work, two kinds of pristine Co-MOF materials were prepared according to the published method with some improvements (Zhao et al., 2015; Sun et al., 2020).

### Preparation of the Modified Electrodes

First, 0.3 and 0.05 μM alumina slurries were used to polished the bare GCE (3 mm in diameter), until it shows mirror-like luster. Second, the electrodes were ultrasonicated in ultrapure water, ethanol, and ultrapure water. Last, nitrogen steam was used to dry the electrodes. A volume of 5 μl (2 mg/ml) of crystal ink of 2D Co-MOFs and 3D Co-MOFs were dropped on the GCE surface (the modified electrode was labeled as 2D Co-MOFs/GCE and 3D Co-MOFs/GCE), respectively. Then, the electrodes were air-dried at room temperature. After that, HAuCl_4_ (1.0 mM) and H_2_PtCl_4_ (1.0 mM) were mixed with the different volume ratio. The Co-MOFs/GCE was immersed in the mixed HAuCl_4_ and H_2_PtCl_4_ solution. AuPt NPs were grown on the surface of the modified electrodes by electrodepositing at −0.2 V (vs. Ag/AgCl). Last, two pristine Co-MOF–supported electrodes were fabricated, and these modified electrodes were washed by ultrapure water.

### Measuring H_2_O_2_ Secreted From Hela Cells

In the electrochemical experiments at the cellular level, 10 mM phosphate-buffered saline (PBS) with N_2_-saturating (pH 7.0) was utilized as the electrolyte to collect the living Hela cells. The details of Hela cells culturing condition were listed in the Supporting Information. With the help of cyclic voltammogram (CV) measurement and amperometric I-t experiment, the redox characteristics of the fabricated electrodes Au_3_Pt_7_/2D Co-MOF/GCE were fully approved and the I-t experiment potential was kept at −0.5 V.

Before the analyzing experiments, the cultured cells were gently washed twice with PBS. Then, 3 ml of PBS was added into the Hela cells culture dish, and 400 μM ascorbic acid (AA) was injected into the dish to stimulate the cells releasing H_2_O_2_. With the amperometric technique, the current response for H_2_O_2_ detection was recorded.

## Results and Discussion

### Characterization of Noble Metal Nanostructures

**FIGURE 1:**
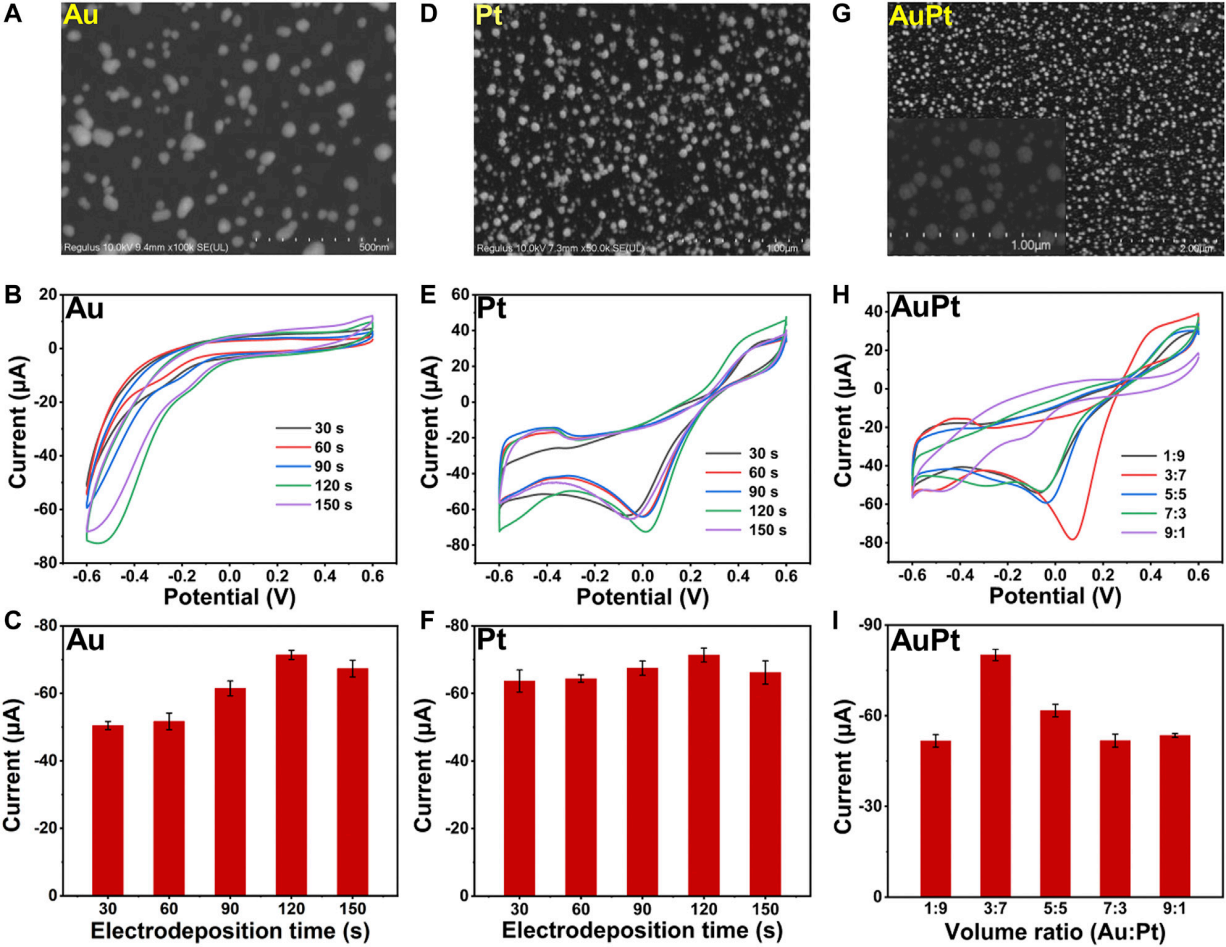
**(A)** SEM images of Au NPs. **(B,C)** The CV curves and histograms of reduction peak current responses of Au/GCE for 2 mM H_2_O_2_ with different electrodeposition time (t = 30, 60, 90, 120, and 150 s) (*n* = 3). **(D)** SEM images of Pt NPs. **(E,F)** The CV curves and histograms of reduction peak current responses of Pt/GCE for 2 mM H_2_O_2_ with different electrodeposition time (t = 30, 60, 90, 120, and 150 s) (*n* = 3). **(G)** SEM images of Au_3_Pt_7_ NPs. **(H,I)** The CV curves and histograms of reduction peak current responses of Au_3_Pt_7_/GCE for 2 mM H_2_O_2_ with different volume ratio of Au NPs : Pt NPs (1:9, 3:7, 5:5, 7:3, and 9:1).

Here, we fabricated a bimetallic electrochemical sensor on the basis of Au_3_Pt_7_ NPs for H_2_O_2_ detection by CV measurements. Scanning electron microscopy (SEM) were employed to characterize the Au NPs, Pt NPs, and Au_3_Pt_7_ NPs. Figure 1A showed the SEM image of Au NPs that dispersedly immobilizing on the electrode. The Au NPs were of spheroidal nature, the average diameter of Au NPs was almost 20 nm. As recorded in the CV curves of Figure 1B, with different time of electrodeposition, the Au NP–modified electrodes showed an increased catalytic ability to H_2_O_2_, and the best catalytic response was obtained at 120 s. Figure 1C exhibited the histograms of the reduction peak current value of different electrodeposition time (t = 30, 60, 90, 120, and 150 s). The detection signal of H_2_O_2_ increased following the electrodeposition time, increasing until the electrochemical signal achieves a peak value of 120 s. Then, the current signal declined with continually increasing the electrodeposition time. The SEM image of Pt NPs is shown in Figure 1D, and the Pt NPs were of spheroidal nature, showing an average size of ∼50 nm. Figures 1E,F demonstrate the CV curves with different electrodeposition time of Pt NPs. The peak current increased from 30 to 120 s and dropped down until 150 s. The result demonstrated that, with the electrodeposition time increased to 120 s, the detection signal of H_2_O_2_ reduction achieved a peak value. Therefore, 120 s was chosen to be the suitable electrodeposition time of synthesized Au_3_Pt_7_ NPs. As shown in Figure 1G, the SEM image of Au_3_Pt_7_ NPs was distributed sparsely on the surface of GCE electrode, where a uniform NP structure with nearly 100 nm exists. Figures 1H,I depict the volume ratio of AuPt NPs (3:7), presenting the best catalytic ability to H_2_O_2_ with the different volume ratio of AuPt NPs (V:V = 1:9, 3:7, 5:5, 7:3, and 9:1). Au_3_Pt_7_ NPs was selected as the optimum nanozyme for fabricating the novel electrochemical sensors. From the results above, metal NPs all gain high catalytic abilities to H_2_O_2_, especially the Au_3_Pt_7_ bimetallic NPs, and the phenomenon may come from the several reasons as follows: 1) electrodeposition provided a considerable route for its low cost, immediate modification, stable current signal, and simple construction; and 2) Au_3_Pt_7_ NPs combining the advantages the high catalytic ability of the Pt NPs and the stability of Au NPs. Therefore, the electrochemical catalytic capability and the selectivity of Au_3_Pt_7_ have been improved (Sun et al., 2017; Yu et al., 2018).

### Characterization of Pristine 3D and 2D Co-MOF

**FIGURE 2:**
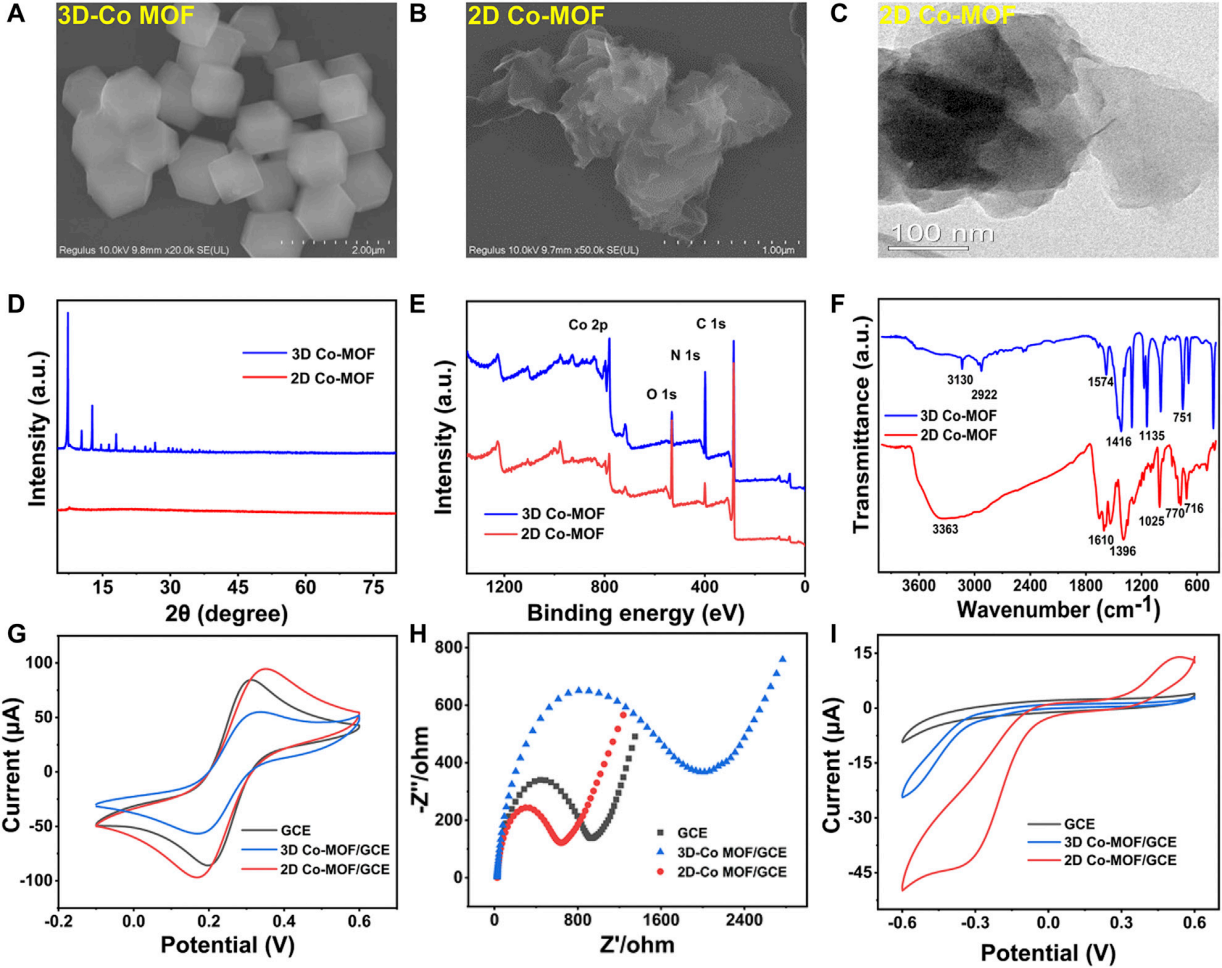
SEM images of **(A)** 3D Co-MOF and **(B)** 2D Co-MOF. **(C)** TEM images of 2D Co-MOF. **(D)** XRD patterns of 3D Co-MOF and 2D Co-MOF. **(E)** XPS survey scan of 3D Co-MOF and 2D Co-MOF. **(F)** The FTIR spectra of 3D Co-MOF and 2D Co-MOF. **(G,H)** CV curves and EIS curves of GCE, 3D Co-MOF/GCE, and 2D Co-MOF/GCE in 5.0 mM K_3_Fe (CN)_6_/K_4_Fe (CN)_6_ solution containing 0.1 M KCl (amplitude 10 mV, impedance spectral frequency 0.1∼10^5^ Hz). **(I)** CV curves of GCE, 3D Co-MOF/GCE and 2D Co-MOF/GCE in 100 mM PBS solution containing 10 mM H_2_O_2_ at a scan rate of 100 mV/s.

The morphology analysis of 2D and 3D Co-MOF was investigated by SEM, transmission electron microscopy (TEM), powder X-ray diffraction (XRD), X-ray photoelectron spectroscopy (XPS), and Fourier transform infrared (FT-IR) spectroscopy. As Supplementary Figure S1 shown, the collected products of pristine Co-MOF exhibited a powder-like 3D Co-MOF that was purple and 2D Co-MOF that was brown, matching with the previous report. Figure 2A shows the SEM image of 3D Co-MOF, which displays a 3D structure rhombic dodecahedron crystalline morphology with the uniform size of nearly 500 nm. Figure 2B reveals the 2D Co-MOF with a sheet-like structure. The crumpled and wrinkled surface indicates its ultrathin property. The TEM image supported 2D Co-MOF with a thin nanosheet structure and confirmed the structure shown in the SEM image. Figures 2C,D reveal the XRD patterns of the 3D Co-MOF and 2D Co-MOF. Figure 2E shows the chemical composition and states of the Co-MOF. As indicated from the two lines of XPS data, there were two main peaks of approximately 780 and 796 eV for 3D and 2D Co-MOF products, respectively, corresponding to Co 2p_3/2_ and Co 2p_1/2_ that are derived from Co^2+^. The two broad peaks at 786.0 and 802.8 eV were satellite peaks, matching well with the reported data. Then, for further investigating of the prepared pristine MOFs, FT-IR spectroscopic studies are shown in Figure 2F, and the characteristic absorption peak of 3D Co-MOF and 2D Co-MOF nanosheet appeared at nearly 716, 770, and 1,025 cm^−1^. These three peaks can prove the existing of skeleton vibration absorption peak of the porphyrin ring (Bai et al., 2019). The peak approximately 1,396 cm^−1^ is related to the stretching vibration of C=N. Besides, the peaks at 1,128 and 1,610 cm^−1^ are generated by the vibration of the benzene ring skeleton outside the pyrrole ring. The absorption peak at 1,700 cm^−1^ can be attributed to the C=O vibration of the carboxyl group on the benzene ring. These results demonstrated that the pristine Co-MOF products had been successfully synthesized.

The examination of CV and electrochemical impedance spectroscopy (EIS) characterized that the pristine Co-MOF of two morphologies was decorated on the surface of the surface of GCE. The electrochemical study of the fabricated sensor in 5 mM K_3_Fe (CN)_6_/K_4_Fe (CN)_6_. As shown in Figures 2G,H, the electrochemical performances of the interfacial of the modified electrodes have been tested by CV and EIS. For the bare GCE, a couple of reversible redox peaks was observed, and the current signals of 3D Co-MOF and 2D Co-MOF–modified electrodes (2D Co-MOF/GCE) were decreased. Moreover, the diffusion-limited process of the electrons was showed in the Nyquist plots. The semicircle diameter displaying the charge transfer resistance (R_ct_) of the modified GCE electrodes. The picture of EIS showed the bad conductivity of the pristine 3D Co-MOF. The Rct of 2D Co-MOF/GCE was close to 800 Ω, and the Rct of 3D Co-MOF/GCE was close to 1,700 Ω. Three-dimensional Co-MOF performed worse than the bare GCE in electrical conductivity properties. Without interlayer interactions and electron confinement, 2D Co-MOF performed better than 3D Co-MOF and bare GCE. As shown in Figure 2I by using the CV technique, the electrocatalytic activity of the selected two pristine Co-MOF for H_2_O_2_ reduction was evident, indicating the current signal of the 2D Co-MOF/GCE closed to 50 μA, the 3D Co-MOF/GCE closed to 23 μA, and the bare GCE was 8 μA. These results illustrated that Co-MOF materials had little influence in the catalytic activity for H_2_O_2_ reduction in 100 mM PBS solution containing 10 mM H_2_O_2_, which can be ignored after the noble metal depositing on the Co-MOF–modified electrodes.

### Characterization of Au_3_Pt_7_ NP-Modified Co-MOF Electrodes

**FIGURE 3:**
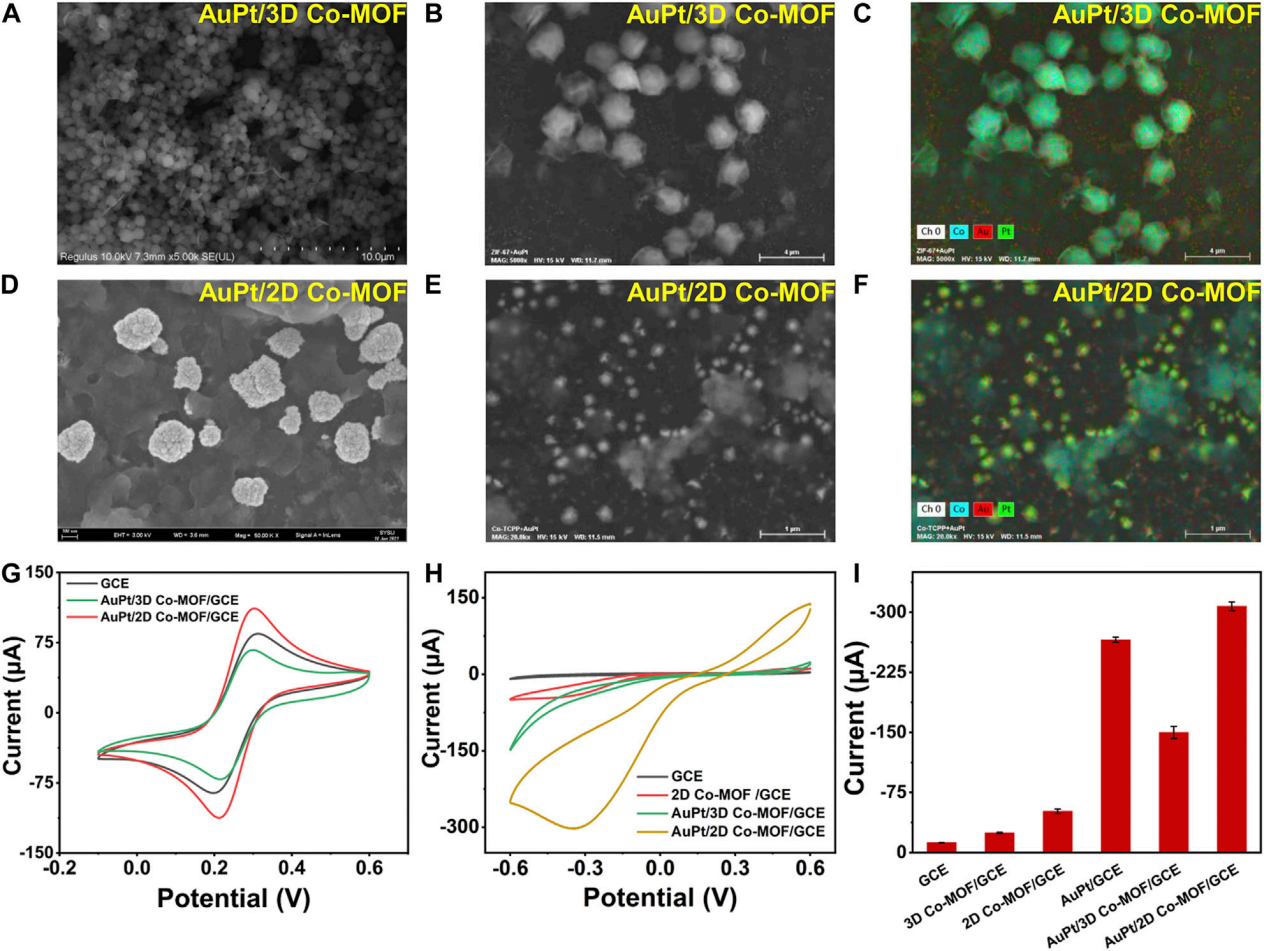
**(A,B)** SEM images of 3D Co-MOF. **(C)** EDS mapping analysis of Au_3_Pt_7_/3D Co-MOF/GCE. **(D,E)** SEM images of Au_3_Pt_7_/2D Co-MOF/GCE. **(F)** EDS mapping analysis of Au_3_Pt_7_/2D Co-MOF/GCE. **(G)** CV curves of GCE, Au_3_Pt_7_/3D Co-MOF/GCE, and Au_3_Pt_7_/2D Co-MOF/GCE in 5.0 mM K_3_Fe (CN)_6_/K_4_Fe (CN)_6_ solution containing 0.1 M KCl (amplitude 10 mV, impedance spectral frequency 0.1∼10^5^ Hz). **(H)** CV curves of GCE, 2D Co-MOF/GCE, Au_3_Pt_7_/3D Co-MOF/GCE, and Au_3_Pt_7_/2D Co-MOF/GCE in 100 mM PBS solution containing 10 mM H_2_O_2_ at a scan rate of 100 mV/s. **(I)** Histograms of GCE, 3D Co-MOF/GCE, 2D Co-MOF/GCE, Au_3_Pt_7_/GCE, Au_3_Pt_7_/3D Co-MOF/GCE, and Au_3_Pt_7_/2D Co-MOF/GCE in 0.1 M PBS containing 10 mM H_2_O_2_.

After figuring out the optimum volume ratio of Au_3_Pt_7_/GCE for H_2_O_2_ detection, the influence of the supporting materials was investigated. Electrodepositing Au_3_Pt_7_ NPs on Co-MOF/GCE, the morphology of Au_3_Pt_7_/3D Co-MOF/GCE and Au_3_Pt_7_/2D Co-MOF/GCE was observed by SEM measurements. Moreover, the CV measurement results exhibited that 2D Co-MOF performed better in supporting that Au_3_Pt_7_ NPs showed the best electrocatalytic activity to reduce H_2_O_2_. As Figures 3A,B reveal, Au_3_Pt_7_ NPs immobilized on the 3D Co-MOF sparsely with an average size of 100 nm. The corresponding EDS spectrum verified the existence of Co, Au, and Pt elements, as shown in Figure 3C and Supplementary Figure S2. The pictures of SEM clarified that the Au_3_Pt_7_ NPs located on the 2D Co-MOF/GCE uniformly (Figures 3D,E). The result of EDS element analysis clearly characterized the Au_3_Pt_7_ NPs combining on the nanosheet 2D Co-MOF. The Co, Au, and Pt elements can be observed distinctly in Figure 3F and Supplementary Figure S3. The result supported that the Au_3_Pt_7_/2D Co-MOF/GCE was synthesized successfully. The small Au_3_Pt_7_ NPs attached better to the 2D Co-MOF nanosheet layers than these were attached to 3D Co-MOF. The redox peak current was increased due to the excellent conductivity of the Au_3_Pt_7_ NPs.

Operating in the solution of 5 mM K_3_Fe (CN)_6_/K_4_Fe (CN)_6_, in Figure 3G, after Au_3_Pt_7_ NPs depositing on two morphologies of pristine Co-MOF–modified electrodes, the gap between the cathodic and anodic peaks of the CV curves has been narrower, and the peak current of Au_3_Pt_7_ Co-MOF/GCE and Au_3_Pt_7_ 2D Co-MOF/GCE was increased. Comparing with the conventional 3D Co-MOF, the 2D Co-MOF nanosheets possessed a larger surface area, smaller diffusion barrier, and more accessible active sites for the substrate molecules (Li et al., 2018). As shown in Figure 3H, compared with the peak current of the pristine 2D Co-MOF–modified electrode, the current signal of the Au_3_Pt_7_/Co-MOF/GCE prominent increased, indicating that the H_2_O_2_ can be catalyzed effectively by the presence of Au_3_Pt_7_ NPs. Preliminarily, the 2D Co-MOF with the better affinity for locating the Au_3_Pt_7_ NPs was inferred. Two-dimensional MOF nanosheets possess unique advantages, such as their large surface area can facilitate the contact of substrate molecules with the active sites on their surface with minimal diffusion barriers (Huang et al., 2017), thus improving their performance in catalysis and sensing applications (Wang et al., 2016). Figure 3I illustrates the peak current of 10 mM H_2_O_2_ to electrodes modified by different materials. The sequence of the peak current increases as follows: GCE < 3D Co-MOF/GCE < 2D Co-MOF/GCE < Au_3_Pt_7_/3D Co-MOF/GCE < Au_3_Pt_7_/GCE < Au_3_Pt_7_/2D Co-MOF/GCE. The sequence indicated the 2D Co-MOF as supporting materials combining the Au_3_Pt_7_ NPs as the compound nanozymes can help get the best electrocatalytic activity to reduce H_2_O_2_.

These results are mainly attributed by the 2D Co-MOF with a highly porous structure and more accessible electroactive sites for adsorbing more Au_3_Pt_7_ NPs than the 3D Co-MOF. On the basis of the Randled–Sevcik equation, the electroactive surface area of different modified electrodes was calculated Ip = (2.69 × 10^5^) AD^1/2^n^3/2^γ^1/2^C, where Ip is the peak current (A); A refers to the effective surface area of the electrode (cm^2^), D means the diffusion coefficient for [Fe (CN)6]^3-/4-^(6.7 × 10^−6^ cm^2^s^−1^), n is the number of transition electrons of [Fe (CN)6]^3-/4-^ (*n* = 1), γ means the scan rate (V/s), and C is the concentration of the redox reactant (5 × 10^−6^ mol cm^−3^). The effective surface area of Au_3_Pt_7_/2D Co-MOF/GCE was determined to be 0.143 cm^2^, which is nearly 1.5 times higher than that of bare GCE (0.09 cm^2^). However, the effective surface area of Au_3_Pt_7_/3D Co-MOF/GCE was determined to be 0.085 cm^2^, indicating the lousy conductivity of the 3D Co-MOF. Therefore, Au_3_Pt_7_/2D Co-MOF/GCE was selected to carry on the further electrocatalytic performance.

### Electrochemical Performance of Au_3_Pt_7_/2D Co-MOF/GCE

The sensitivity of Au_3_Pt_7_/2D Co-MOF/GCE was verified in 10 ml of PBS solution with different concentrations of H_2_O_2_ (2, 4, 6, 8, and 10 mM) (Supplementary Figure S4A). The reduction peak current increased following the increase of H_2_O_2_ concentrations from 0 to 10 mM. The electrochemical signal was plotted, and a good linear relationship was revealed in Supplementary Figure S4B. These consequences preliminarily illuminated that the fabricated electrode possessed a good response for H_2_O_2_ reduction. Supplementary Figure S5A displays the effect of scan rates and the charge transport behavior of electrode Au_3_Pt_7_/2D Co-MOF/GCE that have been investigated, with the help of the [Fe (CN)_6_]^3−^/^4−^redox probe. The CV curves revealed that both the catholic and anodic peak currents were enhanced with increasing scan rates from 50 to 700 mV/s. Furthermore, the current signal responses exhibited a proportional to the square root of the scan rates (Supplementary Figure S5B). The good linear relationship illustrated fast electron transfer kinetics with a typical diffusion-controlled process.

### Amperometric Measurement of H_2_O_2_

For meeting the sensitivity of demonstrating H_2_O_2_ concentration at the cellular level, the suitable detecting potential of the fabricated electrode Au_3_Pt_7_/2D Co-MOFs/GCE needs to be obtained. The amperometric responses were recorded in different potentials from −0.2 to −0.6 V. With the potential increase, the current responses were enhanced under successive injection of 0.4 mM H_2_O_2_, and the solution was stirred continuously at 250 rpm. The highest response showed in the potentials −0.6 V, but the signal noise of the background currents was too larger to be ignored. On the basis of the above results, −0.5 V was selected as the optimal working potential in the following determination (Supplementary Figure S6).

**FIGURE 4:**
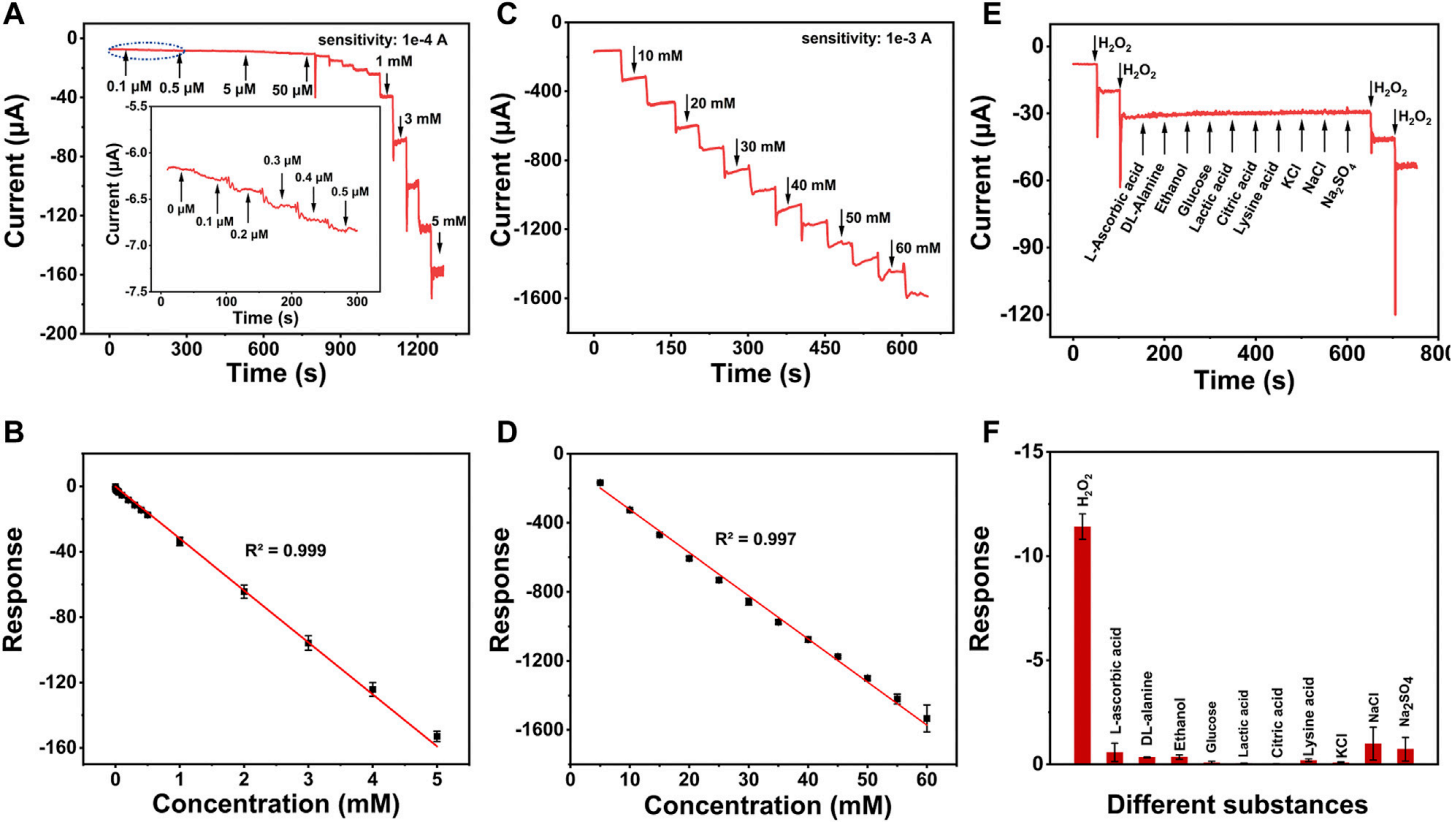
**(A)** Amperometric responses of Au_3_Pt_7_/2D Co-MOF/GCE with the successive addition of low concentration of H_2_O_2_. **(B)** The linear relationships of the response current of Au_3_Pt_7_/2D Co-MOF/GCE with the H_2_O_2_ concentrations from 0.1 μM to 5 mM (R^2^ = 0.999) and **(C)** amperometric responses of Au_3_Pt_7_/2D Co-MOF/GCE with the successive addition of high concentration of H_2_O_2_. **(D)** The linear relationships of from 5 to 60 mM (R^2^ = 0.997). **(E)** Amperometric responses of 5 mM L-ascorbic acid, DL-alanine, ethanol, glucose, lactic acid, citric acid, lysine acid, KCl, NaCl, Na_2_SO_4_, and 0.5 mM H_2_O_2_ in PBS solution at an applied potential of −0.5 V. **(F)** Histograms of different substances.

The amperometric measurement was employed to evaluate the detection sensitivity of Au_3_Pt_7_/2D Co-MOFs/GCE for the reduction of H_2_O_2_. With the successive injection of different H_2_O_2_ concentrations into 10 ml of PBS at the optimum potential of −0.5 V. Figure 4A describes the current response for the low concentration of H_2_O_2_. A typical amperometric plot was exhibited of injecting H_2_O_2_ per 50 s. The insets of Figure 4A show that the current response was achieved at 0.1 μM. The result means that the electrochemical sensor had a good sensitivity response for H_2_O_2_ at a low concentration. Figure 4B reveals the linear regions from 0.1 μM to 5 mM and the linear regression was I (μA) = −31.77C (mM) −0.16695 (R^2^ = 0.999) with the sensitivities of 236.1 μA mM^−1^ cm^−2^. The amperometric plot of 5–60 mM was shown in Figures 4C,D, discovering the liner regression of high concentration of H_2_O_2_, which was I (μA) = −24.98C (mM) −73.26 (R^2^ = 0.997) with the sensitivities of 174.6 μA mM^−1^ cm^−2^ (the sensitivities were calculated by dividing the slope of the linear regression equation by the geometric surface area of the bare GCE). In addition, the low detection limit (LOD) was calculated to be 0.02 μM (S/N = 3). The Au_3_Pt_7_/2D Co-MOF/GCE presented a good electrochemical catalytic activity for H_2_O_2_ with an extended linear range and a lower LOD.

### Selectivity, Reproducibility, and Stability of Au_3_Pt_7_/2D Co-MOF/GCE

The selectivity of Au_3_Pt_7_/2D Co-MOF/GCE for H_2_O_2_ was evaluated by injecting the relevant species successively. As shown in Figures 4E,F, there was no obvious current signal when following adding different substances, including 5 mM L-AA, 5 mM DL-alanine, 5 mM glucose, 5 mM lactic acid, 5 mM citric acid, 5 mM Lysine acid, and 5 mM KCl, NaCl, and Na_2_SO_4_. However, an apparent electrochemical signal was observed when a low concentration of H_2_O_2_ (0.5 μM) was added into the same solvent system. These results indicated that the H_2_O_2_ sensor possessed high selectivity for H_2_O_2_.

The stability of the H_2_O_2_ sensor was investigated. The peak current of CV curves was displayed in Supplementary Figures S7A,B after 7 days, the signal of peak currents only decreased to 93.3%, which presented the excellent stability of the Au_3_Pt_7_/2D Co-MOF/GCE. The same sensor recorded five successive CV measurements. The response current signals shown in Supplementary Figure S8 were almost unchanged with a relative standard deviation of 3.5%, which suggested the excellent repeatability of the electrode. The relevant electrode materials for the electrochemical sensing of H_2_O_2_ displayed in Table 1. The novel electrode Au_3_Pt_7_/2D Co-MOF/GCE exhibited an acceptable electrochemical catalytic activity for H_2_O_2_ with an extended linear range and a lower LOD.

**TABLE 1:** Comparison of the different H_2_O_2_ sensors in detection performance.

Working electrode	Linear range (μM)	LOD (μM)	References
AgAuPt/ITO	4–4,000	2	Peng et al. (2015)
Pt@Au/NWEs	0.15–3.2	0.12	Liu et al. (2017)
ZIF-67-Au@Pt/GCE	0.8–3,000	0.08	Wang et al. (2021)
MoS_2_-Au@Pt/GCE	10–19,070	0.39	Zhou et al. (2017)
PB/Pt@Pd/ITO	0.4–2,247	0.10	Zhu et al. (2019)
Pt@Au/EDA/GCE	1–450	0.18	Li et al. (2013)
Au_3_Pt_7_/2D Co-MOF/GCE	0.1–5,000	0.02	This work
	5,000–60,000		

ITO, indium tin oxide; NWEs, nanowire electrodes; EDA, ethylenediamine (C_2_H_8_N_2_)

### Real-Time Determination of H_2_O_2_ Released From Hela Cells

**FIGURE 5:**
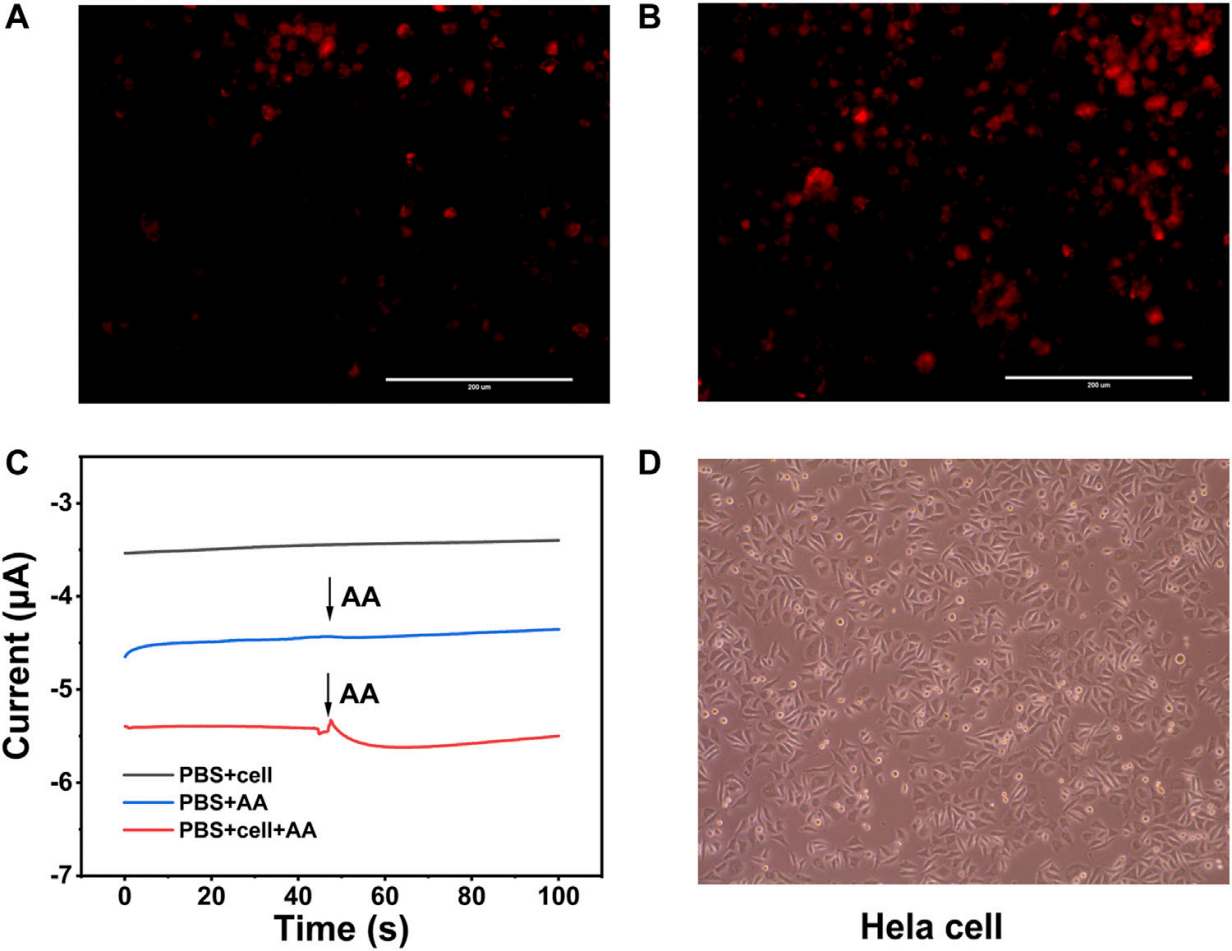
**(A)** DHE fluorescence image of Hela cells without stimulation by AA (400 μM). **(B)** Hela cells stimulated by AA (400 μM). **(C)** Current responses of Hela cells without and with AA (400 μM) stimulations at an applied potential of −0.5 V. **(D)** Bright images of Hela cells after electrochemical detection.

Hela cells have been elected as the model cell to estimate weather the sensitivity of the novel electro-sensor Au_3_Pt_7_/Co-TCPP/GCE could meet the need of evaluating H_2_O_2_ secreted from actual samples. Before the electrochemical detecting, dehydrogenate (DHE) staining was applied for preliminary analyzing the intracellular H_2_O_2_ of Hela cells. As the fluorescence image depicting, the intracellular concentration of H_2_O_2_ was kept at a low level before AA stimulation (Figure 5A). After the treatment with AA (400 μM), the level of endogenous H_2_O_2_ concentration increased apparently (Figure 5B). The DHE fluorescence photographs proved that H_2_O_2_ of Hela cells indeed motivated by AA.

The amperometric technique was utilized to analyze H_2_O_2_ at the cellular level with the potential of −0.5 V. AA was injected into the dish as a stimulant to boost the H_2_O_2_ secreted from the Hela cells. After the injection of 400 μM AA solution into the system without cell, there was a negligible current response. However, an obvious electrochemical current signal was observed when the same drug injected into the system contained the Hela cells at the same concentration. The current value was calculated to be 204 nA, which corresponded to 0.12 μM H_2_O_2_ (Figure 5C). Bright images of Hela cells revealed that the modified electrodes and the electrochemical detection were harmless to the cells, and the morphology and viability of the living cells were similar to those without stimulating (Figure 5D). These results supported that Au_3_Pt_7_/2D Co-MOF/GCE could meet the need of the intracellular H_2_O_2_ *in situ* detection.

## Conclusion

In conclusion, we have fabricated an enzyme-free electrochemical sensor for the real-time detection of H_2_O_2_ released from cells. We discovered that bimetallic Au_3_Pt_7_ NPs showed much higher electrocatalytic activity than pure Au NPs or Pt NPs toward H_2_O_2_ detection. Because of the ultrathin nanosheet structural features, 2D Co-MOF showed a better supporting ability than the 3D Co-MOF. Benefitting from the microplate structures of 2D Co-MOF and the high catalytic ability of Au_3_Pt_7_ NPs, we constructed a novel electrochemical biosensor on the basis of the Au_3_Pt_7_ NPs deposited on the 2D Co-MOF/GCE for the first time. The fabricated Au_3_Pt_7_/2D Co-MOF/GCE exhibited high electrocatalytic activity, fast response, and good sensitivity toward H_2_O_2_ reduction. The linear range of the Au_3_Pt_7_/2D Co-MOFs/GCE provided to H_2_O_2_ appealing in 0.1 μM–5 mM and 5–60 mM and exhibited a LOD of 0.02 μM (S/N = 3). These characters enable the real-time quantification of H_2_O_2_ secreted from Hela cancer cells under drug stimulation. This work provided a new method for improving the detection performance by changing the supporting materials of different crystalline morphologies. For future electrochemical sensors, more efforts will be made in cell adhesion/growth on the surface of the electrodes to get excellent sensitivity *in situ* detection of H_2_O_2_ or other disease biomarkers.

## Data Availability

The original contributions presented in the study are included in the article/Supplementary Material, further inquiries can be directed to the corresponding authors.
