# Supplementary material for: Direct Electrodeposition of Bimetallic Nanostructures on Co-Based MOFs for Electrochemical Sensing of Hydrogen Peroxide
Source: Front Chem. 2022 Mar 11;10:856003. doi: 10.3389/fchem.2022.856003 (PMC8961982; doi:10.3389/fchem.2022.856003)
Supplement: Supplementary file 1 [file DataSheet1.docx]

**Supplementary material**

**Direct Electrodeposition of Bimetallic Nanostructures on Co-Based MOFs for Electrochemical Sensing of Hydrogen Peroxide**

Yixuan Xie ^a^, Xianhua Shi ^a^, Linxi Chen ^a^, Jing Lu ^b^, Xiange Lu ^a,^*,

Duanping Sun ^a,b^*, Luyong Zhang ^a,c,^*

^a^ Center for Drug Research and Development, Guangdong Provincial Key Laboratory of Pharmaceutical Bioactive Substances, Guangdong Pharmaceutical University, Guangzhou 510006, Guangdong, China

^b^ School of Pharmaceutical Sciences, National and Local United Engineering Lab of Druggability and New Drugs Evaluation, Sun Yat-Sen University, Guangzhou 510006, Guangdong, China

^c^ New Drug Screening Center, Jiangsu Center for Pharmacodynamics Research and Evaluation, China Pharmaceutical University, Nanjing 210009, Jiangsu, China

* Corresponding authors.

*E-mail addresses:* lxe_0103@sina.com (X. Lu); sundp@gdpu.edu.cn (D. Sun); lyzhang@gdpu.edu.cn (L. Zhang)

**S1. Experimental Section**

***S1.1. Reagents***

Cobalt nitrate hexahydrate (Co(NO_3_)_2_·6H_2_O), 2-methylimidazole (2-MeIM), methanol (MeOH), hydrogen peroxide (H_2_O_2_), iron(III) chloridehexahyclrate (K_3_[Fe(CN)_6_]), iron(II) chloridehexahyclrate (K_4_[Fe(CN)_6_]), sodium phosphate dibasic (Na_2_HPO_4_), sodium dihydngen phoshate anhydrous (NaH_2_PO_4_), potassium chloride (KCl), sodium chloride (NaCl), Sodium sulphate (Na_2_SO_4_), ascorbic acid (AA), glucose (Glu), citric acid (CA), L-ascorbic acid, DL-alanine, Lactic acid (LA), were all bought from Aladdin Chemistry Co., Ltd. (Shanghai, China). Ultrapure water (18.2 MΩ/cm) was used for all the preparation of solution. The human Hela cells were kindly provided by Professor Jing Lu from Sun Yat-Sen University. All reagents were of analytical grade or better and used as received. Polyvinylpyrrolidone (PVP, average mol wt 40,000), N, N-dimethylformamide (DMF), were purchased from Sigma-Aldrich. Tetrakis(4-carboxyphenyl) porphyrin (TCPP, 97%) was purchased from Tokyo Chemical Industry Co., Ltd. Pyrazine (99%) was purchased from Alfa Aesar.

***S1.2. Apparatus***

Scanning electron microscopy (SEM) and energy dispersive spectroscopy (EDS) images were taken from a field emission scanning electron micrograph (SEM, Geminni500, Germany). Transmission electron microscopy (TEM) images were obtained using a transmission electron microscope (JEM 1400, Japan), which was operated at an accelerating voltage of 200 kV. Fourier transform infra-red (FT-IR) spectra were conducted on a Fourier transformation infra-red spectrometer (IR, EQUINOX 55, Germany). X-ray powder diffraction (XRD) patterns were collected on a PANalytical instrument (Empyrean, Netherlands) to examine the crystal phase of the samples. The surface composition and valence states were analyzed by X-ray photoelectron spectra (XPS, Nexsa, Thermo Fisher Scientific, United States). Fluorescence microscope (Olympus, Tokyo, Japan) was used to observe cells.

***S1.3. Electrochemical measurements***

Electrochemical experiments were conducted with an IGS1230 electrochemical workstation (Guangzhou Ingsens Sensor Technology Co., China) or CHI 660E electrochemical workstation (Shanghai CHI Instrument Co., China) in a traditional three-electrode system. The bare or modified GCE, Pt wire, and Ag/AgCl were employed as the working electrode, counter electrode, and reference electrode, respectively. 0.1 M phosphate buffered saline (PBS, pH 7.0) containing Na_2_HPO_4_, NaH_2_PO_4_, 2.7 mM KCl, and 137 mM NaCl was employed as the electrolyte solution

in all electrochemical experiments for H_2_O_2_ detection. Before experiments, the PBS electrolyte was purged with nitrogen for 15 min to eliminate residential oxygen.

***S1.4.*** ***Synthesis of 3D Co-MOF***

According to the previous literature report, 3D Co-MOF ZIF-67 was synthesized in pure methanol. Co (NO_3_)_2_ 6H_2_O (0.291 g, 1 mmol) and 2-MeIM (0.3284 g, 4 mmol) were separately dissolved in a 30 mL methanol solution. After stirring for 30 min, the solution of Co (NO_3_)_2_·6H_2_O was quickly added into the solution of 2-MeIM. The mixture was stirred for 60 s at 500 rpm. Then it was kept static for 24 h at room temperature. ZIF-67 powder was obtained after centrifuging at 5000 rpm and washing with methanol. Repeating the process three times and dry at 60 ℃.

***S1.5.*** ***Synthesis of 2D Co-MOF***

First, Co (NO_3_)_2_·6H_2_O (44 mg, 0.15 mmol), pyrazine (8 mg, 0.1 mmol) and PVP (200.0 mg) were added into 120 mL mixed solution contained 90 mL DMF and 30 mL ethanol. Second, TCPP (40 mg, 0.05 mmol) was dissolved in 40 mL of the mixed solution of 30 mL DMF and 10 mL ethanol. The TCPP solution was added in the solution we first mixed dropwise under stirring. Finally, the solution was sonicated for 10 min. Heating the final solution to 80 °C for 24 h. Then centrifuged at 8000 rpm and washed twice with ethanol. Drying at 60 ℃ for 24 h, the red nanosheet was obtained.

***S1.6. Cell culture***

Hela cells were grown in Dulbecco's modified Eagle's medium (DMEM, 10%) containing FBS (10%), L-glutamine (4 mM), penicillin (100 units/Ml), and streptomycin (100 mg/mL) under oxidative stress conditions (1 μg/mL lipopolysaccharide (LPS)), and then incubated in a humidified atmosphere of 5% CO_2_ at 37 °C. Hela cells were collected and suspended into sterilized PBS solution (0.01 M, pH 7.4) with specific concentration (cells/mL).

**S2. Supporting figures**


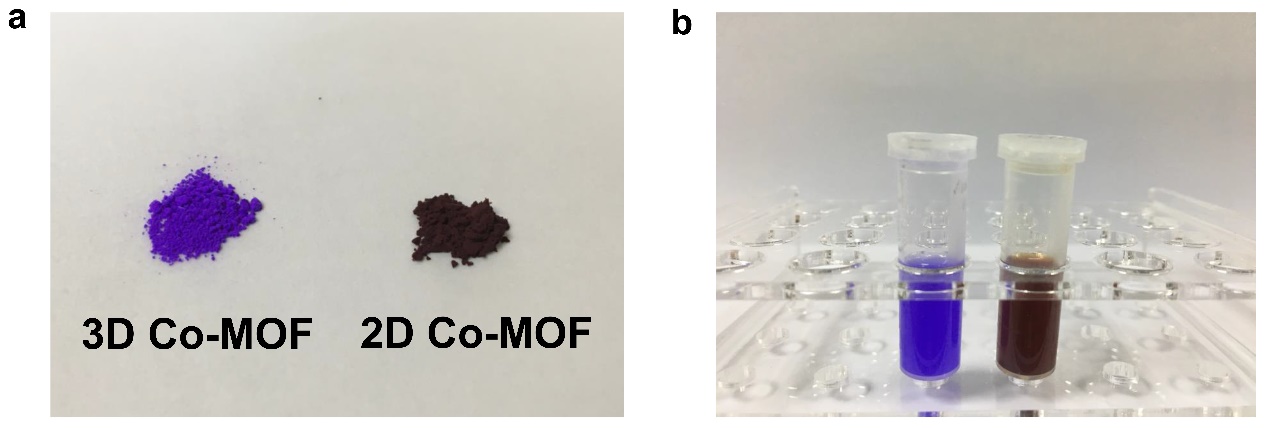


**Figure S1.** (a) The products of pristine Co-MOF and (b) the solution of pristine Co-MOF


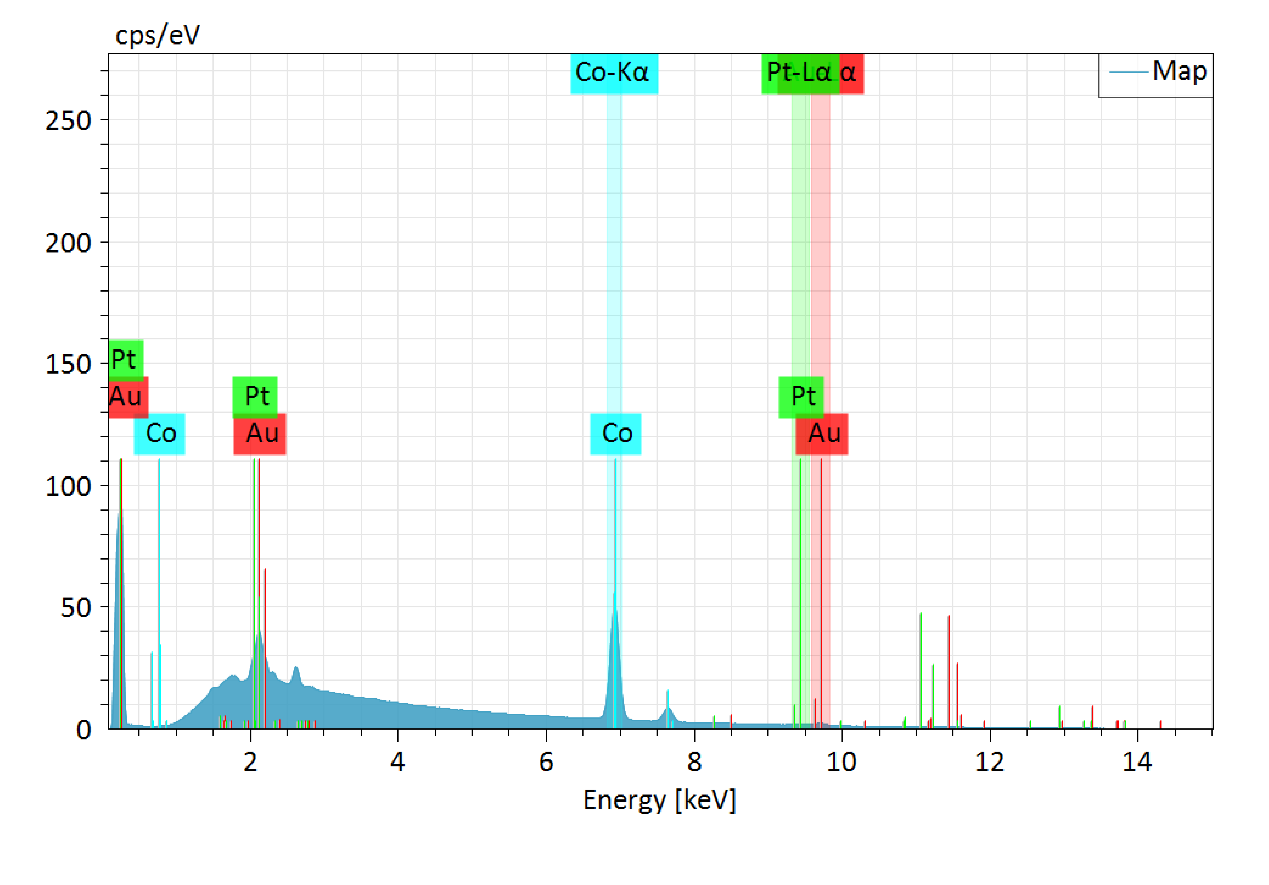


**Figure S2.** The EDS mapping analysis of Au_3_Pt_7_/3D Co-MOF/GCE.


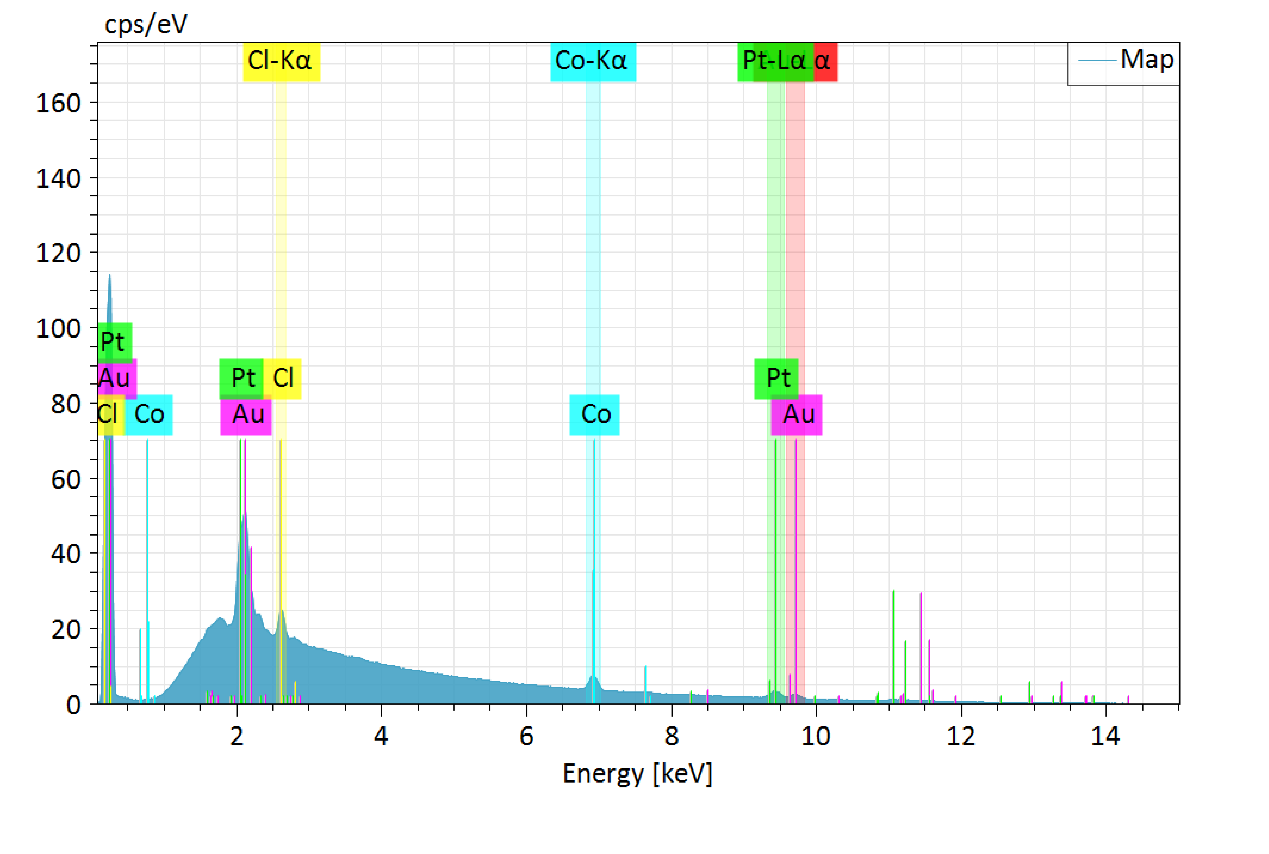


**Figure S3.** The EDS mapping analysis of Au_3_Pt_7_/2D Co-MOF/GCE.


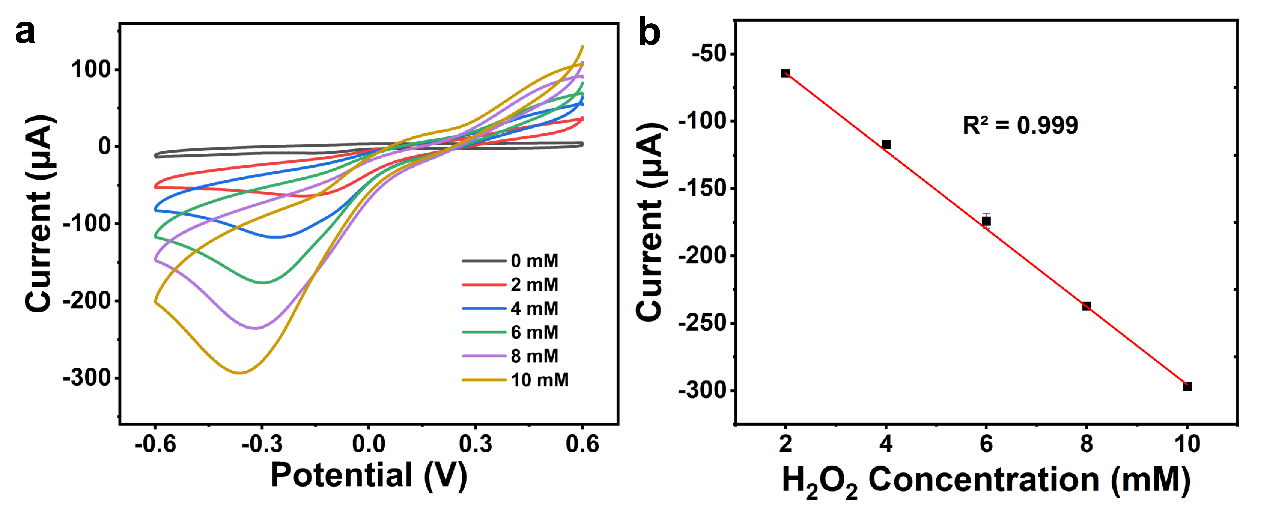


**Figure S4.** (a) CV curves of Au_3_Pt_7_/2D Co-MOF/GCE for different H_2_O_2_ concentrations with a scan rate of 100 mV/s. (b) The linear relationships between the electrochemical peak current vs. H_2_O_2_ concentration (2, 4, 6, 8, and 10 mM).


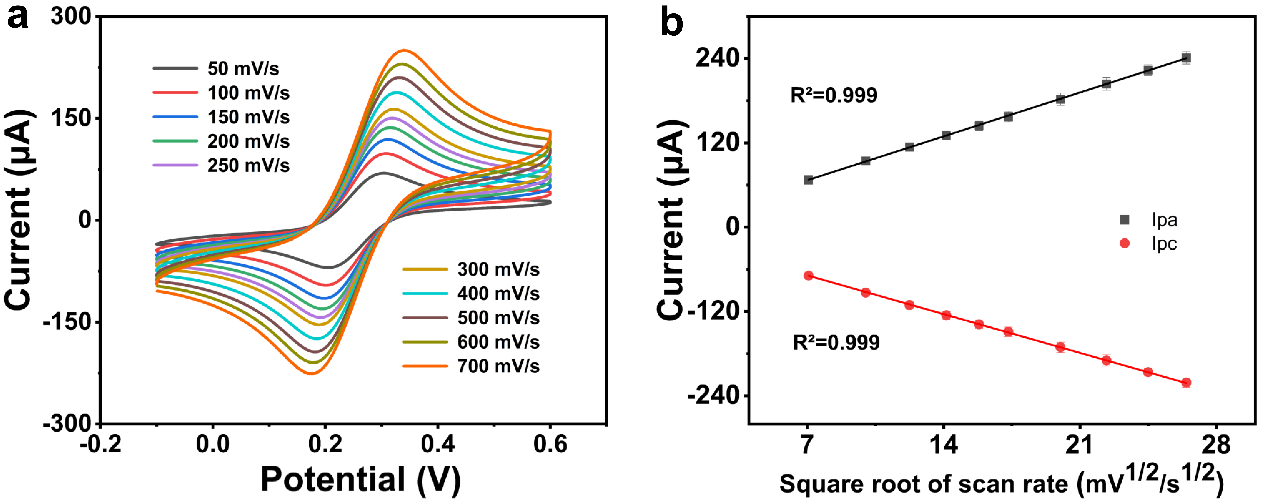


**Figure S5.** (a) CV curves of Au_3_Pt_7_/2D Co-MOF/GCE in 5 mM K_3_Fe(CN)_6_/K_4_Fe(CN)_6_ solution with different scan rates (50, 100, 150, 200, 250, 300, 400, 500, 600 and 700 mV/s). (b) The linear relationships between the electrocatalytic peak current vs. square root of scan rate.


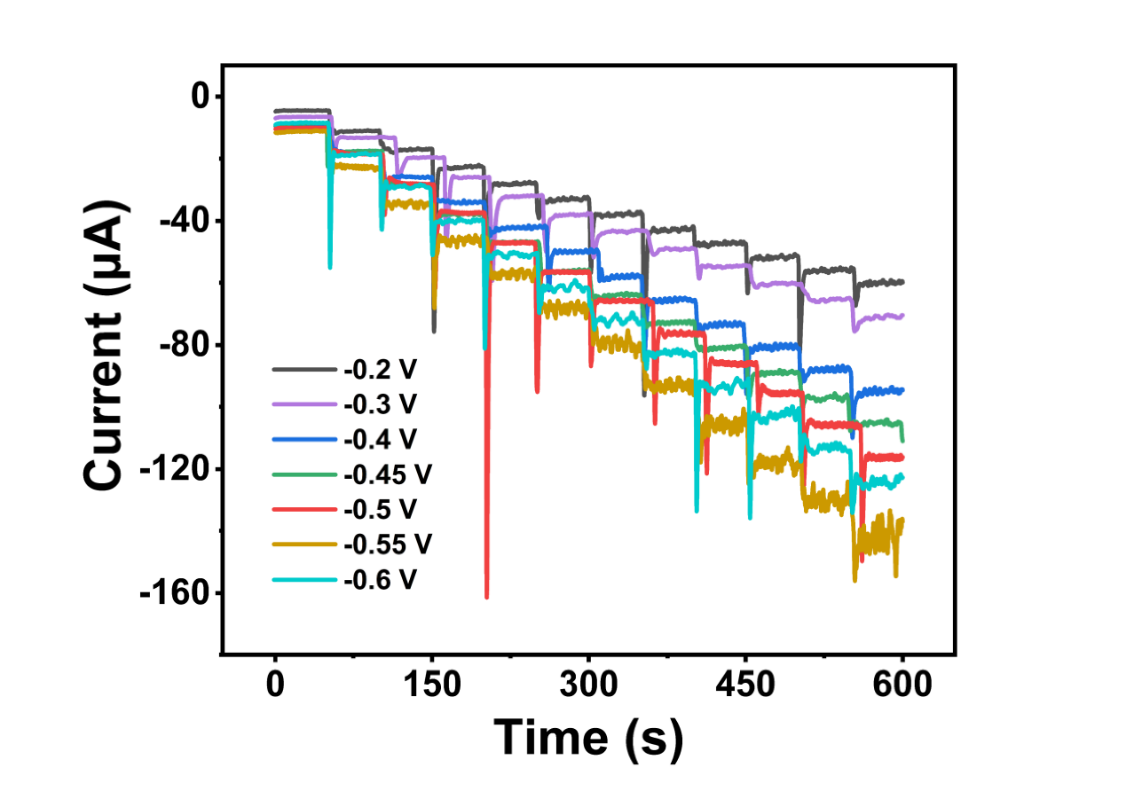


**Figure S6.** Amperometric responses of Au_3_Pt_7_/2D Co-MOF/GCE with different applied potentials (-0.2, -0.3, -0.4, -0.45, -0.5, -0.55, and -0.6 V) by successively injecting 0.4 mM H_2_O_2_ in PBS solution.


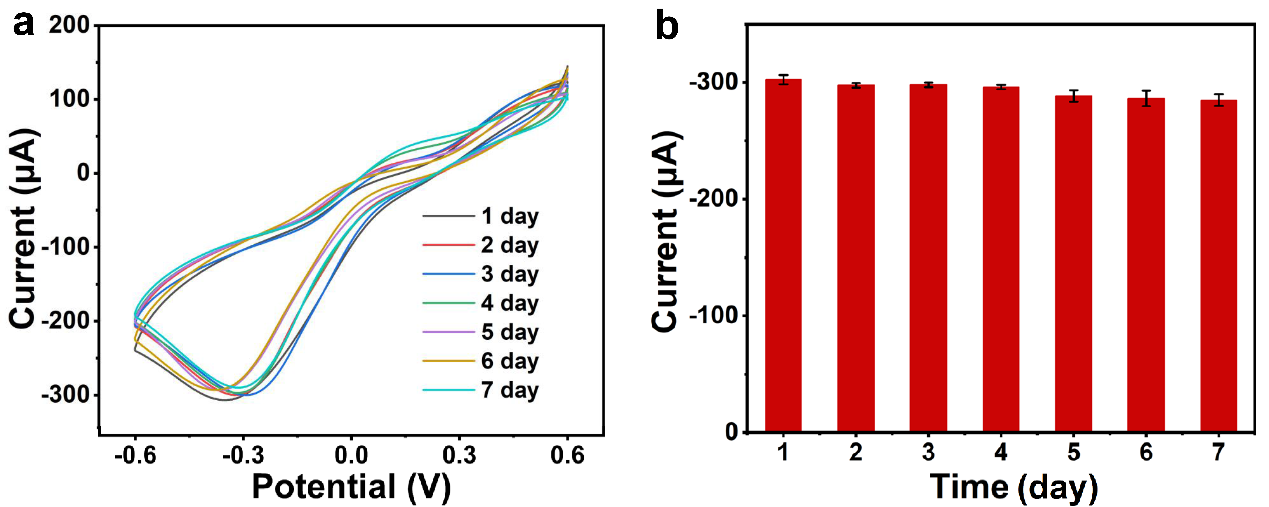


**Figure S7.** (a) CV curves of one day interval until seven days in 0.1 M PBS (pH = 7.0) containing 10 mM H_2_O_2_. Scan rate: 100 mV/s. (b) Current response of Au_3_Pt_7_/2D Co-MOF/GCE toward 10 mM H_2_O_2_ in 0.1 M PBS over 7 days.


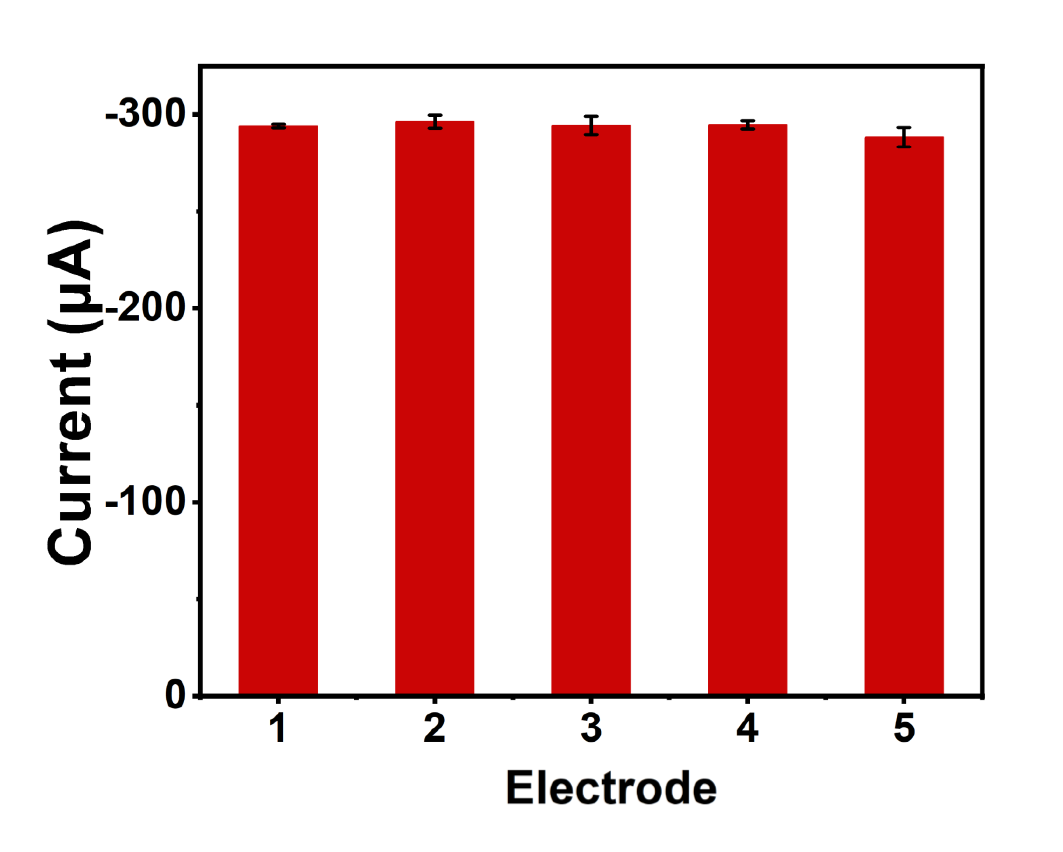


**Figure S8.** Repeatability for five repeated amperometric measurements with the same Au_3_Pt_7_/2D Co-MOF/GCE in 0.1 M PBS (pH = 7.0) containing 10 mM H_2_O_2_. Scan rate: 100 mV/s.
